# The Role of α_1_-Microglobulin (A1M) in Erythropoiesis and Erythrocyte Homeostasis—Therapeutic Opportunities in Hemolytic Conditions

**DOI:** 10.3390/ijms21197234

**Published:** 2020-09-30

**Authors:** Amanda Kristiansson, Magnus Gram, Johan Flygare, Stefan R. Hansson, Bo Åkerström, Jill R. Storry

**Affiliations:** 1 Section for Infection Medicine, Department of Clinical Sciences, Lund University, 221 84 Lund, Sweden; bo.akerstrom@med.lu.se; 2 Division of Hematology and Transfusion Medicine, Department of Laboratory Medicine, Lund University, 221 84 Lund, Sweden; jill.storry@med.lu.se; 3 Department of Clinical Sciences Lund, Pediatrics, Lund University, 221 84 Lund, Sweden; magnus.gram@med.lu.se; 4 Department of Molecular Medicine and Gene Therapy, Lund Stem Cell Center, Lund University, 221 84 Lund, Sweden; johan.flygare@med.lu.se; 5 Department of Obstetrics and Gynecology, Institute of Clinical Sciences Lund, Lund University, 221 84 Lund, Sweden; stefan.hansson@med.lu.se; 6 Department of Clinical Immunology and Transfusion Medicine, Office of Medical Services, 221 85 Lund, Sweden

**Keywords:** α_1_-microglobulin (A1M), red blood cells, hemolysis, heme, Diamond-Blackfan anemia, 5q-minus myelodysplastic syndrome, blood transfusions, intraventricular hemorrhage, preeclampsia, atherosclerosis

## Abstract

α_1_-microglobulin (A1M) is a small protein present in vertebrates including humans. It has several physiologically relevant properties, including binding of heme and radicals as well as enzymatic reduction, that are used in the protection of cells and tissue. Research has revealed that A1M can ameliorate heme and ROS-induced injuries in cell cultures, organs, explants and animal models. Recently, it was shown that A1M could reduce hemolysis in vitro, observed with several different types of insults and sources of RBCs. In addition, in a recently published study, it was observed that mice lacking A1M (A1M-KO) developed a macrocytic anemia phenotype. Altogether, this suggests that A1M may have a role in RBC development, stability and turnover. This opens up the possibility of utilizing A1M for therapeutic purposes in pathological conditions involving erythropoietic and hemolytic abnormalities. Here, we provide an overview of A1M and its potential therapeutic effect in the context of the following erythropoietic and hemolytic conditions: Diamond-Blackfan anemia (DBA), 5q-minus myelodysplastic syndrome (5q-MDS), blood transfusions (including storage), intraventricular hemorrhage (IVH), preeclampsia (PE) and atherosclerosis.

## 1. Introduction

Oxidative stress is defined as an imbalance between the production of oxidants and reactive oxygen/nitrogen species (ROS/RNS), including free radicals, and the antioxidant defense capacity, leading to molecular damage and/or disrupted redox signaling [1]. Many natural processes generate oxidative stress in the body, e.g., microbial defense, cell death and mitochondrial respiration, but it can also be induced by external sources, e.g., UV light, radiation, chemotherapy and air pollution [2]. The contribution of oxidative stress to the pathophysiology of many disorders is well established and may be mediated through, for example, upregulation of stress response genes and oncogenes, generation of mutagen components and inflammation [2].

The main function of red blood cells (RBCs) is to transport oxygen to the tissue. For this purpose, they are packed with hemoglobin (Hb), whereas they lack many other typical cell components such as the nucleus and mitochondria [3]. Each Hb tetramer carries four prosthetic groups, heme groups, which bind oxygen reversibly to the iron atom in the heme group. The iron atom is very reactive and undergoes redox reactions which can result in the generation of ROS. Inside the intact RBCs, cellular protective mechanisms control the ROS generated by Hb, e.g., superoxide dismutase, catalase, peroxiredoxin 2, glutathione peroxidase and glutathione [3]. When hemolysis occurs, the release of Hb and free heme groups results in an increase in the oxidative stress-load, i.e., generation of ROS and/or direct redox reactions with biomolecules, damaging nearby cells and tissue [4]. During normal physiology, however, moderate amounts of leaked RBC components are balanced by an arsenal of protective proteins, a network of endogenous antioxidants, e.g., haptoglobin, hemopexin, heme oxygenases and α_1_-microglobulin (A1M) [4].

In pathological disorders with increased extravascular hemolysis, these defense systems can be reduced or even depleted and, therefore, it has been proposed that supplementation of proteins and antioxidants that can battle the redox imbalance may be beneficial to patients. This review will focus on one of these, A1M, which is a heme- and radical-binding protein with reductase ability [5,6]. Endogenous A1M is found in plasma and extravascular tissue, where it binds heme and radicals and transports them to the kidneys for degradation, thereby acting as a housekeeping protein [7]. The protective functions of A1M have been investigated in a range of cell cultures, organ explants and in vivo models with mitigation of damage generated by oxidative stress as a result.

Recently, our group published data showing that recombinant human A1M, a therapeutic candidate that has been shown to be functionally equivalent to endogenous A1M and that can be produced in large scale, can protect RBCs in vitro and that mice lacking A1M had an altered blood composition [8]. These data suggest that A1M may have a role in RBC development and stability. In light of these new findings, we discuss here the physiological consequences of such a role of A1M in RBC homeostasis by presenting an overview of A1M followed by a discussion of pathological erythropoietic and hemolytic conditions where A1M may constitute a therapeutic opportunity.

## 2. A1M

### 2.1. A1M Protein

**Figure 1:**
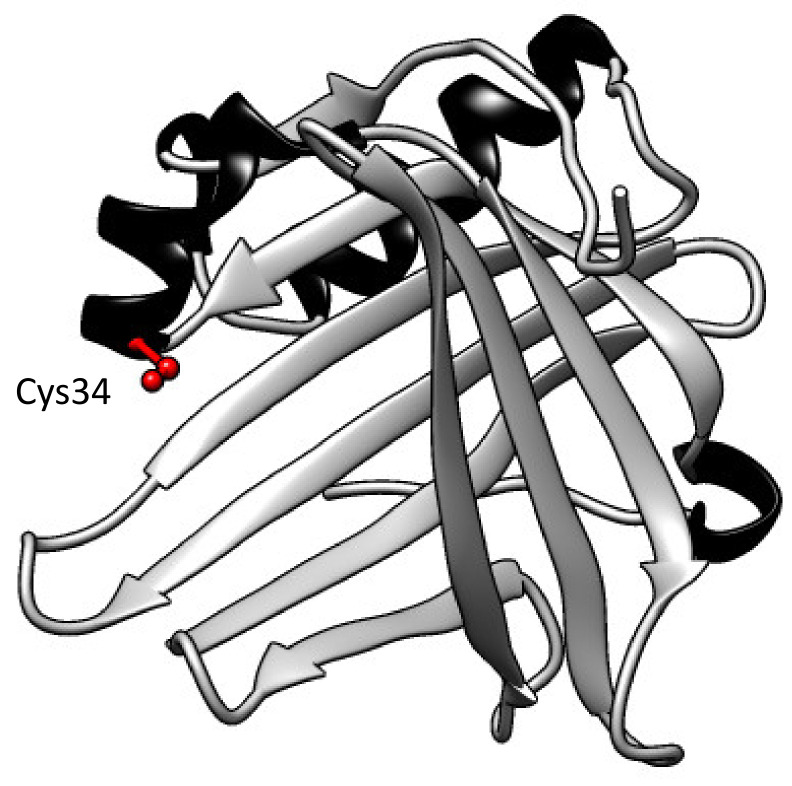
Three-dimensional structure of A1M. The eight anti-parallel β-strands compose a barrel with one open and one closed end. A free solvent-exposed thiol group on pos Cys34 at the open end is marked in red, β-strands in grey and helices in black. Figure was constructed in UCSF Chimera (version 1.14) and is based on the published crystal structure [17].

Human A1M was first discovered and described in the 1970s when it was purified from urine [9]. Subsequently, A1M has been detected in numerous vertebrates, including fish and amphibians [10,11,12,13]. Human A1M is a small glycoprotein with a molecular weight of 26 kDa and with a polypeptide consisting of 183 amino acids [14]. It belongs to the lipocalin protein family and has the characteristic lipocalin folding with eight anti-parallel β-strands that compose a β-barrel with one open and one closed end (Figure 1) [15]. Several different side chains—for example, the free thiol group of Cys34 and three lysyl-residues located around the open end—contribute to the physiologic and protective functions of A1M, such as reductase and radical-binding, described in more detail below [16].

A1M is encoded by the *AMBP* (α_1_-microglobulin-bikunin precursor) gene on chromosome 9, which also encodes the protease inhibitor, bikunin [18]. To date, no common function of these proteins has been described, but they are always transcribed and translated together as a precursor protein. Before reaching the bloodstream, the precursor is proteolytically cleaved into the two separate proteins [19]. This gene construct is conserved in all known species, and, although not fully understood, a recent study of a A1M knock-out model suggests that A1M is important for the folding and post-translational modifications of bikunin [20]. This does not, however, eliminate the possibility that there may be other common functions, both before the proteins are cleaved and/or as a common response to other physiological conditions in the body. *AMBP* expression has also gained attention as a potential diagnostic marker in certain cancers; however, this is most likely connected to bikunin’s role as a protease inhibitor, since the balance between proteases and inhibitors is thought to be important in cancer progression [21,22,23,24].

Transcription of *AMBP* occurs in all nucleated cells and A1M is often referred to as a housekeeping protein. Furthermore, A1M expression is upregulated in the presence of heme, Hb and ROS [25,26,27]. Although not fully mapped out, one suggested stress-induced pathway of transcription of *AMBP* is through the nuclear factor erythroid 2–related factor 2 (Nrf2) pathway. This pathway has been shown to be a central response to oxidative stress, where increased intracellular oxidation results in transcription of cytoprotective proteins [28,29]. In the nucleus, Nrf2 forms a complex with small musculoaponeurotic fibrosarcoma (Maf) proteins which then binds to an antioxidant response element (ARE) in the target genes, including *AMBP* [28,30,31], with subsequent activation of the genes.

**Figure 2:**
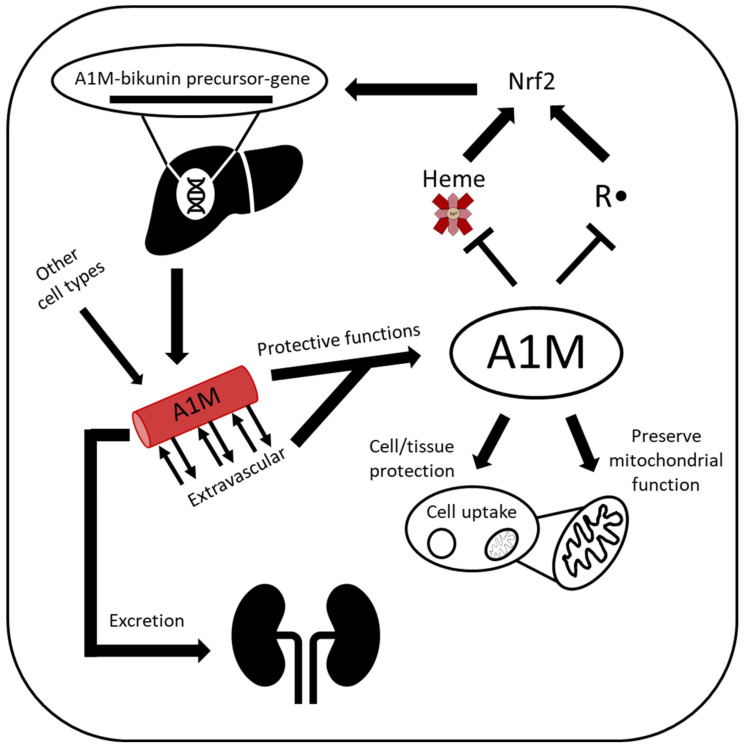
Lifecycle of A1M. A1M is encoded by the *AMBP* gene and is predominantly synthesized in the liver, although most other cell types also express A1M. In the presence of heme and/or oxidative stress, A1M expression is upregulated. A1M circulates in the bloodstream and can also be taken up intracellularly, where it can protect against excessive intracellular oxidative stress and localize to the mitochondria and protect mitochondrial function. A1M is both a radical scavenger with reductase activity and a heme-binding protein and executes these functions to protect cells and tissue before it is cleared by glomerular filtration, followed by tubular reabsorption and degradation.

Most cells express and secrete A1M, including those in the skin, kidneys and placenta, although the primary site of synthesis is the liver [32,33]. From the liver, A1M is secreted into the bloodstream, where it has a short half-life, approximately 2–3 min, before it is extravasated and reaches extravascular compartments [32,34]. In addition to the free form, approximately half of the circulating A1M is complex-bound to IgA, prothrombin and albumin [35]. In the end step of the metabolic route, A1M reaches the primary urine, after filtration through the glomeruli, and is reabsorbed and catabolized in the proximal tubular cells (Figure 2). A small amount, however, is excreted in the urine, and urinary A1M is in fact used as a clinical marker of tubular damage in a wide range of kidney diseases [36,37,38]. Recently, urinary A1M has also been suggested as a biomarker that can help to differentiate between different subgroups in chronic kidney disease and predict kidney injury during hemorrhagic fever with renal syndrome [39,40].

Uptake of both plasma and recombinant A1M has been shown to occur by several different cell types, e.g., kidney cells, blood cells and keratinocytes [8,27,41]. Inside the cells, some A1M localizes to Complex I of the mitochondrial respiratory chain, where it has been suggested to protect mitochondrial function and/or eliminate excessive ROS [27,41]. However, A1M is also internalized by RBCs [8], which lack mitochondria, proposing a general protective function against intracellular oxidative stress.

### 2.2. A1M Protective Properties

The ability of A1M to protect cells and tissue is attributed to various molecular mechanisms: it has been shown to function as a radical scavenger with the potential to both bind and reduce radicals. The interaction between radicals and A1M was studied using the synthetic radical ABTS (2,2′-azino-bis-(3-ethylbenzthiazoline-6-sulfonic acid)) [6]. In addition to reducing the radicals by the Cys34 thiol group, A1M can covalently bind radicals via a trapping mechanism that involves activation of tyrosyl side chains through intramolecular electron migration mechanisms that are initiated by the radical reduction. Thus, a total of 8–9 radicals per A1M molecule are eliminated by reduction and covalent trapping. Furthermore, A1M has been shown to possess catalytic reductase activity, using NADH or NADPH as electron donating co-factors. This reductase activity is rather nonspecific in regard to substrates, i.e., A1M is able to reduce both organic and inorganic compounds, including cytochrome c, metHb, ferricyanide and nitroblue tetrazolium (NBT) [42]. Interestingly, the recombinant forms of A1M have stronger redox properties than plasma and urinary A1M [42]. A possible explanation is that the purified A1M from urine and plasma are partly consumed due to bound chromophores, and these adducts may block the residues involved in the reductase activity, thereby decreasing the activity.

The heme-binding capacity of A1M is conserved in a wide range of species, including human, mouse, bird and fish [43]. This supports the role of A1M as part of the defense network against heme toxicity together with other known heme and Hb scavengers such as haptoglobin, hemopexin and heme oxygenases. Titration of heme established that A1M can bind two heme groups, and the resulting complexes consisted of three A1M and six heme groups (A1M∙heme_2_)_3_ [44]. Furthermore, a truncated form of A1M, called t-A1M, has been shown to catabolize heme [5]. The reductase, radical-scavenging and heme binding properties have been described to constitute different degrees of activity in studies investigating and demonstrating A1M’s broad therapeutic and protective use, described in more detail below.

### 2.3. In Vitro

There are several in vitro studies that demonstrate how A1M employs the different protective mechanisms described above. In the human erythroid cell line K562, the addition of heme, hydrogen peroxide and hydroxyl radicals resulted in increased cytosolic oxidation, which was prevented with the addition of A1M; moreover, silencing of the A1M expression increased the cytosol oxidation [45]. In the same study, A1M prevented heme-induced cell death and cleared cells from bound heme. Both K562 cells and the histiocytic blood cell line U937 upregulated the expression of *AMBP* in response to Hb, heme and ROS [25]. The increased mRNA expression of *AMBP* was also seen in hepatocyte cell line HepG2 in response to oxidative stress and in a primary renal cell line exposed to free heme [25,27].

Radiation is well known to generate the production of ROS [46] and has been shown to cause damage to both directly irradiated cells and non-irradiated bystander cells [47]. Therefore, by reducing oxidative stress, A1M has been suggested to be a potential radioprotector. This was investigated in the study by Olsson et al., where 0.02% of a monolayer culture of HepG2 cells was irradiated with alpha particles, leaving most cells as non-irradiated bystander cells [48]. An increase in apoptotic response genes p21 and p53 and cell death in both directly exposed cells and bystander cells was observed. In addition, there was upregulation of ROS-related cell protection defense genes, among them *AMBP*. Addition of exogenous A1M reduced cell death in the irradiated cells by half and almost completely in the bystander cells. Similarly, the increase in apoptotic and oxidative stress response genes and the cellular oxidative damage, determined by lipid peroxidation and protein carbonyl groups, was reduced with A1M, and the Cys34 residue was described to be central in mediating the protective effect [16,48].

In a recent study, Bergwik and Åkerström provided evidence for a molecular interaction between riboflavin (vitamin B2) and A1M [49]. During exposure of riboflavin to UV light, riboflavin radicals and ROS were formed which resulted in sublethal damage to retinal epithelial cells in vitro, which was counteracted by A1M. Riboflavin was found to be covalently bound to A1M, and the binding resulted in a proteolytic cleavage of the N-terminal of A1M. A1M is upregulated in patients with retinal disease [50] and in skin exposed to oxidative stress [26], suggesting that A1M has a role in tissue exposed to light, combating UV-light-inflicted oxidative stress by binding riboflavin and riboflavin-induced ROS.

The localization of A1M to the kidneys has prompted investigations of its ability to protect kidney cells. Recently, it was shown that A1M decreased the formation of hydroxyl-radicals and heme-induced cell death in the kidney cell line HK-2, using two different forms of recombinant A1M [51]. In a follow-up study, using HK-2 cells and a primary kidney cell line, A1M-reduced cell death and stress gene response was further established, and it was also shown that A1M could protect and preserve mitochondrial function [27]. These results are in line with a previous study where A1M was described to preserve mitochondrial function and structure in several different cell types [41].

### 2.4. In Vivo

Preeclampsia, a pregnancy complication with considerable mortality and morbidity, is a pathological condition in which A1M could potentially be used as a treatment [52]. A1M-mediated protection has been studied in several different in vivo models of preeclampsia [53,54,55,56], and in all cases, a reduction in placental and renal damage was reported. These studies are summarized below in the preeclampsia section. In addition, in a recent publication, A1M was described to confer protection against short-term functional brain damage in an animal model of preterm cerebral intraventricular hemorrhage [57], also further elaborated upon below.

Peptide receptor radionuclide therapy (PRRT) is a cancer treatment modality where intravenously infused radiopeptides bind to tumor cells, which then are killed by ionizing irradiation from the internalized radiopeptides. However, due to the small size of these peptides, they are retained and cleared by the kidneys, resulting in kidney damage in the cancer patients. Therefore, to avoid kidney damage, the doses given to the patients in current clinical practice are limited. A1M has been shown to a have a similar biodistribution and pharmacokinetics to ^177^Lu-DOTATATE, the preferential radiopeptide for treatment of neuroendocrine tumors [58]. In a mouse model of radiation therapy, co-injection of A1M resulted in increased long-term survival and reduced histological and functional renal damage. In the short-term, the mice that received A1M had less DNA damage and upregulation of apoptotic genes in the kidneys [59]. In a follow-up study, it was confirmed that A1M did not interfere with tumor treatment or with the biodistribution of the radiopeptides [60]. Using A1M as a radioprotector may therefore constitute a possibility to improve cancer therapy with PRRT by allowing higher or more frequent radioactivity doses.

Acute kidney injury (AKI) can occur due to a diverse set of underlying conditions such as sepsis, ischemic injury after major surgery, preeclampsia or nephrotoxic drugs [61]. A common denominator in conditions associated with AKI is increased oxidative stress and/or a reduction in the body’s antioxidant defense. In a mouse rhabdomyolysis model, glycerol injections are used to induce the release of muscle cell myoglobin and free heme. This results in subsequent upregulation of cellular protection genes heme oxygenase-1 and heat shock protein-70 in the kidneys, which was shown to be ameliorated by the administration of A1M [51]. Conversely, in a sickle cell disease mouse model of AKI, injected urinary A1M was observed to exacerbate the kidney impairment, which the authors hypothesized was due to A1M binding and transporting heme to the kidneys [62,63]. Although recombinant A1M shows promise as a kidney protector, this study suggests that urinary A1M, with already formed chromophores, may not be an effective treatment and that an overload of both heme and A1M in combination may be unfavorable, even damaging, for the kidneys.

A recently established A1M-knockout (A1M-KO) mouse model elucidated new A1M functions [8,20]. The increased expression of cellular enzymes associated with the unfolded protein response and antioxidants in the livers of A1M-KO mice indicates a role of A1M in the control of the redox environment in the endoplasmic reticulum [20], which in turn is crucial for the protein folding machinery. Furthermore, lower levels of mature, correctly modified bikunin and bikunin complexes were seen in plasma of the A1M-KO mice in spite of significantly higher mRNA levels. This may further explain why bikunin and A1M are co-synthesized, as described above. Interestingly, A1M-KO mice also gained more weight and had the tendency to accumulate fat in the liver [20]. Although other lipocalins have been indicated in appetite regulation [64,65], this has not been described for A1M and may open up new areas within A1M research.

### 2.5. RBC Protection/Homeostasis

A recent article established erythroprotective effects of A1M after investigating different sources of RBCs and various hemolytic insults [8]. RBCs were challenged with heme, hydroxyl radicals or osmotic stress, with additional water resulting in an influx of water to the cells, and in all cases, A1M reduced the resulting cell death. It was shown in vivo that RBCs contained A1M and that RBCs internalized the added recombinant A1M. Although a small protective effect was seen from the internalized A1M, the main reduction in hemolysis was mediated by extracellular A1M. Recombinant human A1M also protected murine RBCs from hemolysis in vitro, suggesting that the effect is not species-specific. These results suggest that the addition of A1M may be beneficial in stabilizing RBCs and reducing hemolysis regardless of hemolytic insult.

Erythrocytes from A1M-KO mice showed a macrocytic anemia phenotype, indicating that A1M is important either for correct development of the RBCs or as an anti-hemolytic protector of the circulating RBCs. Moreover, the erythroid cell line K562 expresses A1M, which may indicate that A1M have a role in protection from oxidative stress during erythropoiesis [25].

A new recombinant version of A1M, rA1M-035, designed to have increased stability and solubility [51], is currently undergoing clinical trials for protection against renal damage resulting from ischemia-reperfusion injury during cardiac surgery. If proven to be effective, as well as maintaining a tolerable safety profile, this would open up new therapeutic possibilities. In the following sections, we describe pathological conditions where RBC lysis and/or Hb- and heme toxicity is a crucial part of the pathological mechanisms, and we thus present possible targets for A1M as a treatment opportunity.

## 3. Erythropoietic Conditions

Diamond-Blackfan Anemia and 5q-Minus Myelodysplastic Syndrome

As mentioned above, A1M protects cells against intracellular oxidative stress caused by excessive accumulation of intracellular heme. A1M may, therefore, have therapeutic potential in disorders where toxic amounts of free intracellular heme contribute to the pathogenic mechanism. Candidate diseases include anemias such as Diamond-Blackfan anemia (DBA) and 5q-minus myelodysplastic syndrome (5q-MDS). In DBA and 5q-MDS, inherited and acquired, respectively, heterozygous inactivating mutations in ribosomal proteins cause impaired ribosome synthesis. The clinical manifestations of anemia and bone marrow failure are likely connected to failing ribosome biogenesis and/or altered mRNA translation capacity in these disorders [66].

An important question is why erythropoiesis is particularly sensitive to a limiting pool of available ribosomes. One hypothesis, recently backed by several publications, states that reduced translation of globin mRNA leads to accumulation of free heme, which contributes to DBA and 5q-MDS pathology [67,68,69,70].

Hb synthesis in erythropoiesis is a particularly heme-dependent physiological process where heme molecules need to be stoichiometrically matched with the globin protein synthesis. Erythroid progenitor and precursor cells therefore require dynamic and exact control of intracellular heme concentration to prevent heme buildup or deficiency, which both cause severe anemia. Examples of heme-regulatory mechanisms include heme-dependent inhibition of heme-regulated eIF2α kinase (HRI). HRI is a kinase that, in the absence of heme (e.g., in iron deficiency), phosphorylates eIF2a, which then reduces mRNA translation and thereby arrests globin protein production. In the presence of heme, however, heme binds and inhibits HRI, which allows uninhibited globin synthesis.

Another essential heme regulator is the heme exporter FLVCR1. In the sequential stages of normal erythroid development, there is a short window where heme is produced in excess of globin and heme export by FLVCR1 is required to protect the cells from heme toxicity [71]. Cats infected with feline leukemia virus-C (FeLV-C), which binds to and interferes with FLVCR1 function, develop profound macrocytic anemia with absence of reticulocytosis and specific depletion of erythroid precursor cells in the bone marrow [71]. FLVCR1-null mice also show a phenotype resembling DBA, with arrested erythroblast maturation, macrocytic anemia and developmental deformities [67].

The phenotypic similarities between DBA and FLVCR mutant mice and FeLV-C-infected cats suggest a common pathophysiology involving heme toxicity. There is a significant induction of free heme and ROS in normal erythroid cells transduced with shRNA constructs targeting the three DBA genes, *RPS19*, *RPL5* and *RPL11* [72]. The increased production of ROS in ribosomal protein-deficient erythroid cells leads to increased expression of *FLVCR1* and decreased expression of *BACH1* [72]. Furthermore, ribosome availability can be considered a part of the heme autoregulatory loop, since free intracellular heme upregulates expression of ribosomal protein genes during early erythropoiesis in order to balance the globin:heme stoichiometry [73].

**Figure 3:**
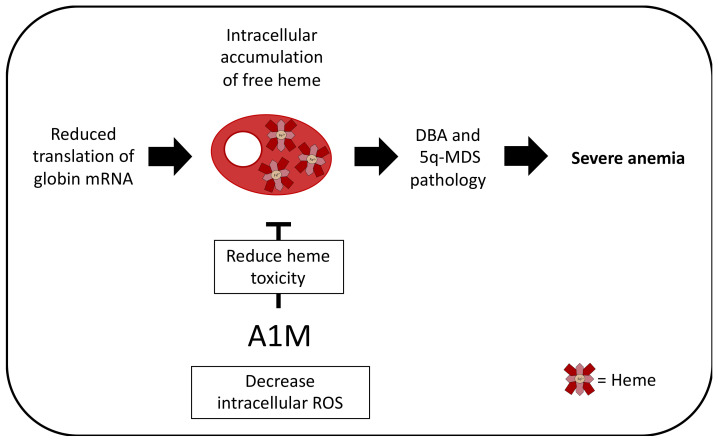
Potential therapeutic effect in DBA and 5q-MDS. In DBA and 5q-MDS, mutations in ribosomal proteins cause impaired ribosome synthesis, leading to the reduction of translation of globin mRNA. Since heme molecules need to be stoichiometrically matched with globin proteins to produce Hb, this leads to the accumulation of free heme within the erythroid progenitor and precursor cells. This contributes to the DBA and 5q-MDS pathology. A1M has been shown to reduce intracellular ROS and heme-associated toxicity and it could therefore be speculated that A1M is a potential therapeutic agent in DBA and 5q-MDS.

While Abkowitz and colleagues have suggested that heme toxicity in DBA and 5q-MDS can be corrected using inhibitors of heme synthesis, a more physiological approach would be to utilize the cytoprotective and antioxidant effects of A1M as a therapeutic agent in DBA and 5q-MDS (Figure 3) [41,74]. To further clarify the role of A1M in protecting erythroblasts in the bone marrow from heme toxicity, it will be important to evaluate if the macrocytic anemia in A1M-KO mice [8] is associated with excess ROS levels in erythroid precursor cells.

## 4. Hemolytic Conditions

### 4.1. Intraventricular Hemorrhage in Preterm Infants

Brain hemorrhage usually occurs as a result of a rupture of a weakened blood vessel or following head trauma. This is followed by the release and accumulation of blood-derived components, mainly erythrocytes and plasma proteins, in the brain parenchyma (intracerebral hemorrhage), the subarachnoid space (subarachnoid hemorrhage) or within the ventricles (intraventricular hemorrhage, IVH), leading to disruption of normal anatomy and increased local pressure. Cerebral IVH is a major complication of prematurity, with an incidence of approx. 25% in very low birth weight infants [75,76]. Despite a significant improvement in neonatal care over the last few decades, preterm infants remain at a high risk of neurodevelopmental disability or mortality following cerebral IVH [77,78].

The etiology of cerebral IVH in preterm infants is multifactorial, complex and heterogeneous. A generally accepted description is that preterm IVH typically originates by vessel rupture in the germinal matrix, a vascularized area with an inherent fragility, but rupture of the choroid plexus has also been described as a site of rupture and bleeding [79,80,81]. Following vessel rupture, blood is rapidly accumulated within the ventricles. Depending on the dynamics of the hematoma expansion, the primary damage occurs within minutes to hours and is mainly a consequence of mechanical damage. The events of secondary brain damage are not fully understood, but the activation of a number of pathways by the presence of extravasated blood in the ventricular space is often described to be central. Deposition of blood is followed by rupture of the erythrocytes and subsequent release of extracellular Hb into the cerebrospinal fluid (CSF). Hb that escapes the intra-erythrocyte compartment is highly reactive and spontaneously autoxidizes to form a range of metabolites, including met- (Fe^3+^) and ferryl hemoglobin (Fe^4+^), free heme, iron and ROS. All of these components are known to be initiators of cytotoxic, oxidative, pro-inflammatory and apoptotic events inducing tissue damage [4,82,83]. In a preterm rabbit pup model of IVH, it has been observed that extracellular Hb and its downstream metabolites cause structural damage of the choroid plexus [84,85,86]. Furthermore, following IVH, extracellular Hb was found to be widely distributed in periventricular white matter and deposited in remote regions of the cerebellar white matter, leading to alteration of normal development of the cerebellar cortex [81,87].

**Figure 4:**
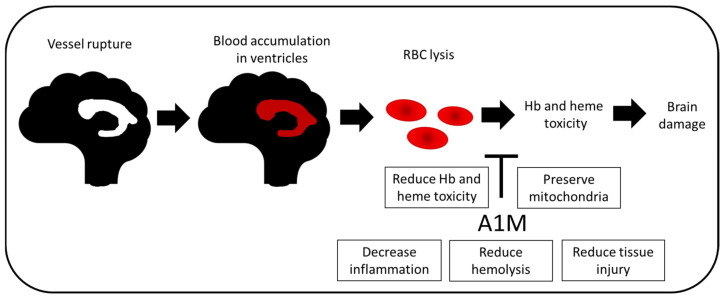
Potential therapeutic effect in IVH. In preterm infants, vessel rupture leads to blood accumulation in the ventricles. This results in mechanical damage to the brain. Deposition of blood and thereafter rupture of RBCs, resulting in release of extracellular Hb into the CSF, causes further damage to the brain. Hb, heme and ROS are pro-inflammatory and cytotoxic molecules which cause tissue and cell damage. Administration of A1M has shown protective effects in the immature brain, with less inflammation and tissue injury as well as preserved mitochondrial function. Moreover, recent studies suggest that prevention of hemolysis and reduced Hb and heme toxicity are potential mechanisms of A1M protection.

At present, there is no available therapy to prevent or treat cerebral IVH in preterm infants. Considering the role of extracellular Hb, free heme and ROS in the development of brain damage, the potential of A1M to confer protection of the immature brain following IVH was recently investigated [57]. Using a preterm rabbit pup model of IVH, it was found that intracerebroventricular (i.c.v.) administered rA1M is widely distributed within the immature brain following IVH. Administered rA1M was found in areas with high plasticity, and interestingly, it appeared to follow the distribution of extracellular Hb, i.e., immunofluorescence labeling of rA1M and Hb displayed a high co-existence. Furthermore, early functional protection was analyzed following i.c.v. administration of human plasma-derived A1M (hA1M) and showed reduced structural damage of the ependymal epithelium as well as preserved mitochondria. Administration of hA1M significantly reduced cellular activation, inflammatory response and tissue injury, suggesting that administration of hA1M blocks the toxic reactions of extracellular Hb-metabolites. In addition, the recently reported anti-hemolytic effect of A1M [8] (see above) suggests that the prevention of hemolysis of RBCs in the ventricles may be an additional protective mechanism of A1M in IVH (Figure 4). Taken together, this warrants further investigation of rA1M/hA1M as a potential candidate for neuroprotective intervention against brain damage following preterm IVH.

### 4.2. Blood Transfusion

**Figure 5:**
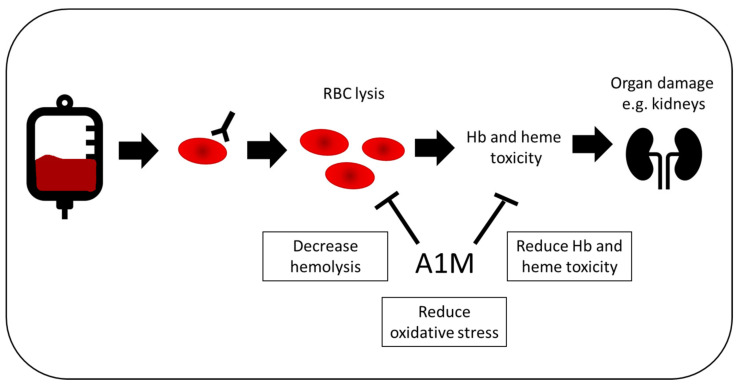
Potential therapeutic effect in blood transfusion. In patients receiving blood, 1–2% will produce antibodies to a blood group, increasing the risk of acute or delayed hemolysis. This is linked to organ damage, such as renal damage, which can be both transient and permanent. A1M could be used as a treatment during blood transfusions, in order to decrease both hemolysis and the resulting oxidative damage of free Hb and heme.

Blood transfusion has long been recognized as a safe and, in many cases, life-saving treatment. Commonly, whole blood donations are processed further into RBC concentrates, plasma and platelet concentrates and transfused according to the patient’s clinical symptoms and needs. Although the majority of transfusions are complication-free, there is a risk of increased hemolysis in instances where a patient produces antibodies to one of the many minor blood group antigens. It is estimated that between 1% and 2% of all patients receiving blood will produce an antibody to a blood group antigen, a figure that has remained constant throughout the years [88,89]. In these patients, there is a risk of acute or delayed hemolysis in the event of transfusion of antigen-positive blood. In the most recent Serious Hazards of Transfusion (SHOT) Report [90], hemolytic transfusion reactions accounted for 1.4% of all incidents (49/3397) reported to the scheme during 2019. Of these incidents, four were acute hemolytic events, four were hyperhemolysis (discussed below) and 30 were delayed hemolytic reactions (≥3 days post transfusion), and it is likely that the latter category is under-reported since patients have often been discharged before the onset of symptoms. Acute and hyperhemolytic episodes are associated with renal damage, which may be temporary or permanent. It has been shown previously that A1M can protect against renal damage following radiation treatment and rhabdomyolysis [59,60]; however, its protective role during active RBC hemolysis is not clear, and Ofori-Acquah et al. have shown a correlation between kidney damage and increased A1M-bound heme in sickle cell disease [62,63]. Thus, the role of A1M protection in hemolytic transfusion reactions needs to be examined further (Figure 5).

Chronic transfusion is also associated with increased hemolysis, especially in sickle cell disease, where antibodies to multiple blood group antigens are not uncommon among transfused patients [91,92]. This increases the risk that the patient will be transfused with incompatible blood due to limitations in finding phenotypically matched blood. Furthermore, these patients are susceptible to hyperhemolysis syndrome, a life-threatening crisis in which both transfused RBCs and their own cells are rapidly destroyed [93,94].

More recently, there has been a return to whole blood transfusion in trauma situations and other instances of massive bleeding [95,96], although the clinical benefits are still under debate. In trauma situations where group O blood is used, there is a potential risk of increased hemolysis due to anti-A and anti-B antibodies present in the plasma. Much focus has been placed on screening for low titer donors to avoid this complication [97]. A1M is potentially a useful prophylactic drug in such circumstances, in order to reduce both hemolysis and the downstream oxidative effects of free Hb and heme.

The storage lesion has been studied intensively over past decades and still it is not completely understood [98,99]. Currently, RBCs may be stored for up to 42 days at 2–8 °C prior to transfusion. Following processing, they are maintained in a preservative solution such as SAG-M or ADSOL that contains glucose, adenine, mannitol and salts to maintain glycolysis. Over time, physiological changes occur due to storage conditions and concomitant decreasing pH that result in modification of the RBC membrane. While there is no international consensus on the minimum required survival, the US Food and Drug Administration mandates that 75% of a compatible unit of blood should survive at 24 h post-transfusion. In other words, this means that up to 25% of all RBCs in a unit can be destroyed or taken out of circulation during the first day. Mays and Hess calculated the median RBC loss due to storage lesion to be 17.6% [100,101]. Not only does this stress the recipient’s capacity to remove dead RBCs but it has also been shown recently that altered glycation end products of transfused RBCs can increase endothelial ROS [102], thus doubling the systemic insult. Therefore, a potential use of A1M could be to protect RBCs from lysis during storage and thus increase the number of live cells for transfusion and reduce endothelial oxidative stress caused by lysed cells in recipients.

### 4.3. Preeclampsia

As stated above, preeclampsia (PE) is a severe pregnancy-related condition that annually affects 8.5 million women worldwide. It is a leading cause of maternal and perinatal morbidity and mortality, responsible for ~18% of all maternal deaths and ~25% of intrauterine growth restriction (IUGR) cases and 40% of neonatal deaths globally [103]. While its etiology remains largely unknown, diagnosis of PE is currently based on the maternal clinical manifestations: high blood pressure (BP) and general organ damage (including IUGR) manifesting after 20 weeks of gestation. Currently, symptomatic BP treatment is the only available treatment and delivery is the only known cure.

Two stages of PE pathogenesis can be distinguished [104,105]. The first stage involves incomplete conversion of the spiral arteries and incomplete, superficial in-growth of trophoblasts into the maternal decidua [106]. This is believed to lead to uneven blood perfusion and subsequently oxidative stress [107]. Evidence suggests that oxidative stress further aggravates the vascular function in the placenta [108], resulting in insufficient blood perfusion, inflammation and tissue damage [109]. It is thought that these events cause a breach in the placental barrier, leading to the leakage of fetal- and/or placental-derived factors into the maternal circulation [110]. Clinical manifestations of the second stage become apparent after 20 weeks of gestation and are likely linked to the general vascular endothelial damage caused by placental-derived material and debris [104].

Increased placental production of fetal hemoglobin (HbF) [111,112] as well as increased maternal plasma levels of HbF and low levels of HbF scavenger proteins have been described to further aggravate the oxidative stress and inflammation seen in PE and IUGR [113,114]. One potential source of HbF is lysed fetal RBCs. In fact, it was recently shown that IUGR babies had higher levels of free Hb and lower heme oxygenase-1 (HO-1) levels [115]. Extracellular Hb, including HbF, and heme are suggested to be inducers of tissue damage in PE [116,117]. Since free Hb is a potent scavenger of the vasodilator nitric oxide [118], it possibly contributes to some of the vasoconstriction and hypertension observed in PE.

In PE, elevated levels of free HbF in the maternal blood are detected as early as the first trimester [119] and in term pregnancy shown to correlate with the BP, i.e., the severity of the disease [120]. In animal models, free HbF causes kidney and placental damage similar to that observed in PE [54,55,56]. Studies using the ex vivo dual placenta perfusion model demonstrated that free Hb causes PE-like damage to the blood–placental barrier and endothelial cells by inducing oxidative stress and inflammation as well as leakage of free HbF over the blood–placenta barrier into the maternal circulation [121].

**Figure 6:**
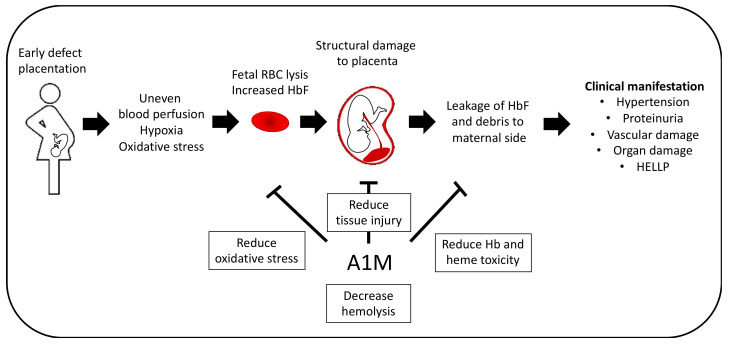
Potential therapeutic effect in PE. PE pathogenesis can be distinguished by two stages. Early defect placentation is believed to contribute to the development of PE by leading to uneven blood perfusion, hypoxia and increased oxidative stress, which potentially results from lysis of fetal RBCs. This leads to structural damage to the placenta and leakage of HbF and debris into the maternal side, where it contributes to the clinical manifestation of PE, the second stage, e.g., hypertension, vascular endothelial damage and proteinuria. A1M have shown promising results in several in vivo studies by reducing tissue injury, oxidative stress and Hb toxicity as well as reduction of hemolysis of fetal RBCs in vitro.

Interestingly, studies have shown that plasma and urinary levels of A1M are elevated in women with PE and IUGR [120], and it has been suggested that A1M plays a part in the defense against both PE and IUGR [122]. The therapeutic effect of rA1M against Hb-induced damage to tissues and organs, such as the placenta and kidneys, has been demonstrated both ex vivo [121] and in pregnant animal PE models [54,55]. Moreover, A1M has, together with HbF, been suggested to be a biomarker of PE [119]. More recently, A1M was also shown to reduce hemolysis of fetal RBCs [8], a potential source of HbF that may contribute to the clinical manifestation of PE. In this study, it was also seen that the fetal RBCs were also more sensitive to oxidative stress than the adult RBCs, which may be due to their lower antioxidant capacity [123,124]. This antihemolytic effect may contribute to the potential protective effect and further supports the notion that A1M may be used as a treatment in PE (Figure 6).

The most severe manifestation of PE is eclampsia, characterized by general seizures caused by swelling of the central nervous system. In a proteomic study, elevated levels of free Hb were shown in the CSF of preeclamptic women [125]. Recently, Van der Berg et al. demonstrated increased levels of A1M, particularly in women with neurological symptoms [126], again suggesting a natural neuro-protective function of A1M, similar to that seen in the therapeutic strategy for IVH described above.

The HELLP syndrome (**H**emolysis, **E**levated **L**iver enzymes, **L**ow **P**latelet count) is a severe form of PE that occurs in 10–20% of cases of severe PE [127]. As with PE, delivery is the only known cure. For women in earlier gestational weeks (24–34), when stabilizing the mother and, if required, giving treatment for fetal lung maturation [127], one might speculate that A1M could potentially be used as a co-treatment to ameliorate progression of the disease by protecting the kidneys and the vascular endothelium, reduce hemolysis in women with HELLP syndrome, prevent eclampsia and/or as a potential therapy for IUGR.

### 4.4. Atherosclerosis

Atherosclerosis, a cardiovascular disease, is one of the leading causes of deaths in developed countries [128]. It is defined by a thickening of the intimal layer and accumulation of fat leading to the build-up of a plaque/atheroma in the vascular system. The more advanced plaque is covered by a fibrous cap and contains a necrotic core with, e.g., fat and cholesterol [129].

Vulnerable plaques with thin fibrous caps are prone to rupture and can lead to myocardial infarction or stroke [130,131]. One of the characteristic features that precedes these acute ischemic events is intraplaque hemorrhage (IPH) [132]. Neovascularization is increased in unstable plaques. The neovessels are immature, fragile and leaky, which leads to extravasation of RBCs within the plaques: IPH [133]. The RBCs entering the necrotic core lyse and promote inflammation, oxidative stress and cholesterol deposits from the membranes [134]. As discussed above, extracellular Hb from RBCs results in toxicity to the surrounding cells and tissues, which now is believed to be an important part of disease progression in atherosclerosis, with IPH being linked to plaque progression and ruptures [133].

Statins are the prevailing pharmacological treatment today. Statins lower the cholesterol content of the RBC membranes and Tziakas et al. showed that statin treatment reduced intraplaque hemorrhage and, therefore, subsequent addition of cholesterol crystals to the plaque core [135]. In a recent study, however, half of the study participants showed sub-optimal response to statins [136]. Therefore, there is an urgent need for new treatment strategies. HO-1 has been suggested as a potential therapeutic target (reviewed in [137]). HO-1 binds and degrades heme intracellularly, and the resulting degradation product bilirubin has antioxidant effects [138]. HO-1 deficient mice have accelerated plaque formation in atherogenic models [139], and, contrastingly, mice with overexpressed HO-1 tend to have slower plaque formation [140]. This indicates that heme toxicity plays a significant role in atherosclerosis progression.

A1M was suggested to have potential in targeting heme toxicity in atherosclerosis by Jeney et al. in 2014 [134], proposing that it would battle atherosclerosis by targeting the heme toxicity and resulting oxidative stress. Moreover, the recent finding of RBC stabilization [8] could also reduce the IPH, leading to less plaque ruptures.

Myeloperoxidase (MPO), a heme-containing enzyme present in granules in the neutrophil, has been identified in atherosclerotic lesions. MPO has been shown to oxidize LDL and thereby contribute to the progression of atherosclerosis [141]. In a study published in 2015, MPO-A1M interactions resulted in proteolytic cleavage of A1M into the heme-degrading t-A1M and, moreover, A1M was shown to reduce oxidation of LDL by MPO in the presence of hydrogen peroxide [142].

**Figure 7:**
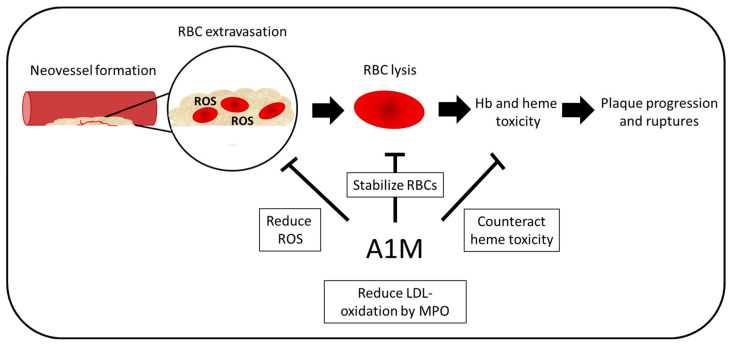
Potential therapeutic effect in atherosclerosis. In atherosclerosis, neovascularization is increased in unstable plaques. These vessels are often leaky, which leads to RBC extravasation within the plaques. Due to high presence of ROS, the RBCs lyse, which leads to increased oxidative stress and, therefore, further plaque progression and ruptures. A1M could have multiple protective functions including battling heme toxicity and ROS as well as stabilizing the RBCs. Moreover, LDL oxidation by MPO, a feature known to contribute to the progression of atherosclerosis, which is inhibited by A1M.

Furthermore, the recently described A1M-KO mice gained more weight and showed fat accumulation in the liver [20], which are both atherosclerotic risk factors [143,144,145]. Although plaque formation and vascular health were not examined in the A1M-KO mice, this suggests that A1M may play a role in protection against atherosclerosis. In summary, published data suggest that A1M could inhibit both the oxidation of LDL and thereby the progression of atherosclerosis and counteract the damaging reactions of extracellular Hb-metabolites after IPH (Figure 7). Therefore, battling heme toxicity and ROS, as well as stabilization of extravasated RBCs, with A1M could be a novel treatment strategy for atherosclerosis. However, further investigations into oxidized LDL levels and the use of A1M-KO as a disease model are needed in order to provide insights into A1M’s potential protective role and as a potential treatment in atherosclerosis.

## 5. Conclusions

The reductase and heme- and radical-binding protein A1M has been shown to have therapeutic effects in several in vitro and in vivo models of pathological conditions that result from oxidative insult. In light of new findings regarding the antihemolytic effects of A1M, here, we discuss additional erythropoietic and hemolytic conditions where A1M’s heme- and radical-binding, reductase and antihemolytic abilities could interact and be utilized as a potential treatment.
